# The Role of Stress Experience and Demographic Factors for Satisfaction with Life in Norwegian Adolescents: Cross-Sectional Trends over a Ten-Year Period

**DOI:** 10.3390/ijerph20031940

**Published:** 2023-01-20

**Authors:** U. K. Moksnes, S. T. Innstrand, M. Lazarewicz, G. A. Espnes

**Affiliations:** 1 Department of Public Health and Nursing, NTNU Center for Health Promotion Research, Norwegian University of Science and Technology, 7030 Trondheim, Norway; 2 Department of Psychology, NTNU Center for Health Promotion Research, Norwegian University of Science and Technology, 7030 Trondheim, Norway; 3 Department of Health Psychology, Medical University of Warsaw, 02-091 Warsaw, Poland

**Keywords:** wellbeing, life satisfaction, youth, adolescence, normative stressors, interpersonal stressors, school stressors

## Abstract

Background: The individual’s perception of life satisfaction (LS) is regarded as a key indicator of one’s overall experience of wellbeing, sensitive to the broad spectrum of functioning. Adolescence is particularly an important period for assessing LS and factors associating with LS. The present study investigated cross-sectional trends in adolescents’ LS levels across three time points over a 10-year period, as well as the role of stress experience and socio-demographic differences in association with LS. Methods: The study used cross-sectional data from three time points: 2011 (n = 1239), 2016 (n = 1233), and 2022 (n =311), including adolescents from lower and upper secondary public schools, with an age range of 13–20 years. Results: There were relatively high and stable mean scores on LS across all time points; however, significant differences were found between 2011 and 2016. Results from the multivariate linear regression analysis showed that sex and age were moreover weakly associated with LS, where LS decreased slightly between the ages of 13 and 18 years and increased from 19 to 20 years. Of the stress domains, interpersonal and school-related stressors showed the strongest negative and significant association with LS; significant interaction effects of sex by stress domains were found, but not with sex by time. Conclusion: This study supports the relatively high and stable level of LS in adolescents across the investigated time points. Demographic factors were moreover weakly associated with LS. The findings also contribute by showing the significant role of interpersonal and school-related stressors in association with LS.

## 1. Background

In today’s society, it is acknowledged that assessments of the populations’ quality of life (QoL) and subjective wellbeing (SWB) are essential for understanding the welfare of individuals and progress of society [1,2]. This is in addition to measuring welfare aspects such as access to education and work, freedom, and material resources [3,4]. The concept of SWB includes people’s appraisals and evaluations of their own lives, including cognitive judgements, such as life satisfaction (LS) and experiences of positive and negative emotions [5,6]. LS represents the cognitive component of SWB and refers to an individual’s cognitive, global appraisal of life as a whole [5,7]. Unlike emotional responses, which may fluctuate, overall appraisals of LS have been shown to be a more stable indicator of SWB and for examining adolescents’ perceptions of their global life circumstances and functioning [6,7]. 

Questions about what factors contribute to change and variation in LS have been debated [1]. Adolescence is a distinct period related to understanding variations in LS because of the significant changes, transitions, and adaptions in virtually every aspect of an individual’s life (physical, social, and psychological). However, studies on LS have mainly been conducted on adults or young adults, with relatively limited research on the adolescent population [5,6]. The transition period from childhood to adulthood now occupies a greater portion of the life course than ever before, due to the earlier onset of puberty, paralleled with the delayed timing of adult responsibilities. The age range between 10 and 19 years has commonly been used to describe the age of adolescence [8]. However, recently, the age range of 10–24 years has been suggested to be more appropriate, reflecting the present understandings of this life phase and is used to define the sample of school students aged 13–20 years old in this study [8]. Consistent with findings reported in studies with adults, most studies with adolescents show that they report high LS, especially in high-income Western countries such as Norway [9]. Regarding trends in self-reports of mental health and wellbeing, an increase in LS was found in a study on the Norwegian adult population from 1984 to 2008, especially for the older age group (40–69 and 70+ years) [1]. A related study based on the same material showed that a significant proportion of the responders had a long-term within-person LS change over the 20-year period [2]. Norwegian studies have shown a strong increase in the prevalence of symptoms of depression and anxiety among young people, especially among adolescent girls [10,11,12]. A Nordic study (which included Norway) investigating the prevalence of high LS in adolescents aged 11–15 years old between 2002 and 2014 indicated large changes in the prevalence levels at the country level [9]. Norway showed an overall increasing prevalence of high LS over the 12-year period observed, especially in the youngest age group [9]. Studies in Western European countries have shown variations in the levels of LS and wellbeing in the younger age groups, showing stability or weak declines in LS and wellbeing over time [13,14,15]. The relationship between demographic factors (sex, age) and LS seems to be moderately strong [6]. A few studies have shown that LS and wellbeing decrease with age [9,16], whereas other international studies (European, Mexican, and Nordic) have shown that LS seems to be more stable [14,17], or even increases with age, during adolescence [18]. Moreover, girls seem to score lower on LS than boys [8,13,16] and show a larger decline in LS during the adolescent years [9,16]. 

An individual’s perception of LS is regarded as a key indicator of one’s overall experience of life circumstances, sensitive to a broad spectrum of functioning [7]. One important factor that may have an impact on LS during adolescence, based on the changes and transitions occurring during this life phase, is the experience of stress. In the present study, stress is defined as a subjective experience of the condition that results when person–environment interactions lead the individual to perceive a discrepancy—whether real or not—between the demands of a situation and the resources available to the person to cope adequately [19]. Stressors signify situations and pressures that cause stress. In addition to major life events that may affect adolescents more randomly, exposure to potential minor chronic or normative everyday stressors (family, peers, and school) increases during the adolescent years and may affect adolescents’ ability to cope, consequently affecting wellbeing [14,20,21,22]. Relevant stressors during adolescence include increasing academic demands, school–leisure conflict, as well as interpersonal stressors including getting along with peers, parents, and romantic relationships [20,21,22]. In this context, boys seem to experience lower levels of stress than girls [23,24]. Research on the relation between experience of stressors and LS is limited. However, previous research has showed a negative association between LS and stress related to school [20], family [25], and general normative stressors [21,22]. LS has been found to be positively related to a broad spectrum of positive personal, psychological, behavioral, social, interpersonal, and intrapersonal outcomes [5,26]. Although few studies have used the term “stressors” in association with LS, young people’s negative evaluations of academic/school variables and interpersonal variables could be perceived as potential stressors in adolescents’ lives. Adolescents with higher LS also seem to report more positive psychosocial functioning, compared with those with lower LS [5,6,26]. 

Adolescence is a particularly important period for understanding levels and trends in LS due to the changes and transitions occurring during this life period, both personally and contextually. A study of changes and transitions may contribute with valuable information to understanding changes in LS [6]. Although the link between stress and health outcomes is well established, few studies have investigated the role of different stressor domains, including school, family, and peers, in association with positive outcomes such as LS. These aspects call for investigating (a) cross-sectional trends in levels of LS, as well as (b) the role of stress domains and socio-demographic differences in association with LS. This study investigated:

(1) Variations in the average level of LS among adolescents aged 13–20 years old including three cross-sectional samples from 2011, 2016, and 2022;
(2) The associations between LS and time, sex, age, and stress domains, controlled for self-rated health;
(3) The interaction of sex × time, and sex × stress domains in association with LS.

## 2. Methods

### 2.1. Procedure

The study was based on cross-sectional data from a study called ‘Oppvekst i bygder’ (Living in rural communities) where data have been collected every five years since 1996. This study used data from three time points (2011, 2016, and 2022), and included adolescents from lower and upper secondary public schools. The data from 2011 and 2016 included five municipalities, and the data from 2022 included four municipalities in Mid-Norway. Teachers administrated the questionnaires during a 45 min classroom session where the students could either choose to answer the questionnaire or do schoolwork. An information letter was sent to all students and parents of those under 16 years of age. Students ≥16 years old gave consent to participate by answering the questionnaire, whereas written parental consent and consent from students were obtained for students 13 to 15 years old. The study procedures were approved by the Regional Committee for Medical Research Ethics Mid-Norway.

### 2.2. Participants

#### 2.2.1. 2011 Sample 

In total, 1924 students (total number in the enrolled schools) were invited to participate in the study, and n= 1239 responded, with an age range of 13–18 years (response rate 64%). The sample included 634 (51.2%) girls and 603 (48.7%) boys; 2 respondents did not identify their sex (Table 1).

#### 2.2.2. 2016 Sample

In total, 1906 students (total number in the enrolled schools) were invited to participate in the study, and 1282 completed the questionnaire (response rate of 67%). The age range of the included sample was restricted to 13–20 years, reducing the sample size to n = 1233. In the sample, 580 (47%) were girls and 644 (52.2%) were boys; 9 respondents did not report their sex (Table 1).

#### 2.2.3. 2022 Sample

In total, 1538 students (total number in the enrolled schools) were invited to participate in the study, and 311 completed the questionnaire (response rate 20.2%). The sample consisted of 167 (53.7%) girls and 141 (45.3%) boys; 3 respondents did not identify their sex (Table 1). The age range of the sample was 13–20 years. 

### 2.3. Measures

#### Demographic Variables Included Sex and Age

Life Satisfaction (LS) was measured using the 5-item Satisfaction with Life Scale (SWLS) [7,27]. The SWLS assesses the cognitive dimension of subjective wellbeing rated on a seven-point Likert scale, ranging from (1) strongly disagree to (7) strongly agree. A higher total score indicates higher LS (min 5, max 35). The SWLS has been used extensively and found to be appropriate for assessing LS in both adults and adolescents [7]. The Cronbach’s alpha value for the present study was 0.87. 

Stress was assessed using the Norwegian 30-item version of the Adolescent Stress Questionnaire (ASQ-N) [28]. The ASQ is designed to measure normative stressors that adolescents may experience in their daily life and the extent to which the stressor experience has constituted a psychological challenge for them. Items are rated on a five-point Likert scale, ranging from (1) not at all stressful or is irrelevant to me to (5) very stressful; a higher score indicates a higher stress level. The scale consists of seven dimensions covering stress related to: school performance (e.g., item: Having to study things you do not understand), school/leisure conflict (e.g., item: Not enough time to have fun), peer pressure (e.g., item: Being hassled for not fitting in), home life (e.g., item: Abiding by petty rules at home), romantic relationships (e.g., item: Making the relationship work with your boyfriend/girlfriend), teacher/adult interactions (e.g., item: Not being listened to by teachers), and school attendance (e.g., item: Abiding by petty rules at school) [28,29]. The ASQ has been evaluated in different samples of European adolescents, indicating adequate psychometric properties [30,31]. Cronbach’s alpha values for the sub-scales are presented in Table 2.

Self-rated health was assessed by one item, “How is your health at the moment?” The response options were: (1) very bad, (2) bad, (3) neither good nor bad, (4) good, and (5) very good. Measuring overall subjective health among adolescents using one item has previously been used in other studies on adolescents and found to be a valid indicator of overall health [32].

### 2.4. Statistical Analyses

Statistical analyses were conducted using SPSS 27.0 and Stata version 17. Descriptive statistics including means and standard deviations were calculated for the scales in the study. Multiple linear regression analysis was used to investigate associations between sex, age, time point, stressor domains, and the criterion variable LS, controlled for self-rated health. Self-rated health was included in the regression model because it is a potential confounder in association with both stress [33] and LS [34]. Differences in the levels of LS according to time point were investigated with dummy variables, where the year 2011 was used as the reference category. When looking at the stressor domains, each domain was investigated separately in association with LS in the unadjusted and adjusted multivariate regression model. Interaction effects were tested with interaction terms including sex × time and sex × each of the stress domains. Effect size for the multiple regression model was calculated using Cohens’ f^2^ with values of 0.02, 0.15, and 0.35 indicating small, medium, and large effect sizes, respectively. The proportions of missing values for the variables of stress, self-rated health, and LS varied in the range of 2.3–5.2%. In the construction of scale sum scores, cases with missing responses at a proportion of 20% or less were included. Model assumptions for linear regression analysis were tested, with no indications of multicollinearity. The VIF values for the independent variables ranged between 1.02 and 2.93, and the average VIF was 1.80. VIF ≥ 5 to 10 indicate multicollinearity among the variables in the regression model [35]. The Breusch–Pagan test is used to test for heteroskedasticity in a linear regression model and assumes that the residuals are normally distributed. The test indicated heteroscedasticity; however, no serious violations were found because of the large sample size. The scatter plot showed a random pattern of residuals. Multivariate linear regression analysis was conducted with a listwise deletion of cases. *p*-values ≤ 0.05 were considered statistically significant.

## 3. Results

### 3.1. Variations in the Average Level of LS among Adolescents

Table 1 shows the distribution of demographic characteristics. The distribution of sex was quite equal across the three measurement points, whereas the distribution of age groups varied more across time points. When looking at the total mean scores on LS across time points (Table 2), the highest score was in 2016, followed by 2022, and the lowest was in 2011. When looking at the mean scores on LS for sex (Table 2), they were all above the neutral point of the scale (≥20) at all time points, where boys reported higher scores than girls. When looking at the age groups, the highest mean scores on LS were in the age group of 13–14-year-olds at all time points, followed by the age groups 15–16 years old and 17–20 years old. The mean stress scores were moderately high across all three time points; however, the stressors related to school performance, school/leisure conflict, and peer pressure showed the highest scores. When looking at self-rated health, the mean scores were above the neutral point of the scale at all time points. 

### 3.2. Associations between LS and Time, Sex, Age, and Stress Domains, Controlled for Self-Rated Health

Table 3 presents the results from the multiple linear regression analysis for associations between sex, age, time point and stress domains and the criterion variable LS. When looking at time, those participating in 2016 reported significantly higher LS than those in 2011 in the unadjusted model (β = 0.12) and in the model adjusting for sex and age (β = 0.16). No significant difference in LS was found between 2011 and 2016 when controlling for self-rated health and stress domains (β = 0.04). No differences were found in LS between timepoint 2022 and 2011 in the unadjusted or adjusted results. Sex was significantly associated with LS in both the unadjusted model (β = 0.14) and when controlling for age (β = 0.14), where boys scored higher than girls. However, no sex differences were found when controlling for time, stress domains, and self-rated health (β = 0.03). Age showed a negative curvilinear association with LS in all the regression models, where levels of LS declined from 13 to 18 years and increased from the age of 19 to 20 years. The results showed that all stressor domains were significantly and negatively associated with LS in all the models; however, the strongest associations were found for stress of peer pressure (β = −0.27) and home life (β = −0.26), followed by school performance (β = −0.23) and school attendance (β = −0.23), controlled for sex, age, time, and self-rated health. 

### 3.3. Interaction Effects of Sex × Time, and Sex × Stress Domains in Association with LS

When looking at the interaction effects between sex × time and sex × stressor domains, there were significant interactions between sex × peer pressure, sex × home life, sex × school attendance, and sex × school performance, with stronger associations for girls (Table 3). The interaction between sex and time was not significant. 

## 4. Discussion

This paper furthers our understanding of the mean levels and trends in adolescents’ report of LS over three time points, as well as the association between sex, age, time, stress domains, and LS in adolescents. Three findings stand out in this study: (a) the generally stable level of LS across the three time points investigated, although significant differences in LS scores were found between 2011 and 2016; (b) the significant but modest role of demographic factors in association with LS; and (c) the significant negative association between stress domains and LS with interaction effects of sex by stress domains found.

Similar to previous findings [5,6], the descriptive results presenting mean scores on LS were in the positive range, with scores above the neutral point of the scale (mean score ≥ 20) at all three time points; the highest score was in 2016. Multivariate results from the linear regression analysis showed that time was significantly associated with LS, with a significantly higher score found for adolescents in 2016 compared with those in 2011 when controlling for sex and age. However, no significant differences were found when controlling for self-rated health and stress domains, which is the main result to be interpreted. No differences in LS were found between 2022 and 2011. The findings are in line with previous studies on adolescents showing generally high mean scores on LS, varying between 23.0 and 25.0 [27]. The present findings contribute to the understanding that LS is perceived as stable by adolescents across time points investigated and are interesting to compare with the Nordic study [9], which showed high levels of LS, especially in Norwegian adolescents 11–15 years old from 2010 to 2014; however, variations in levels of LS were found among the different countries compared. The present findings correspond with studies among adolescents in high-income countries showing small declines or stable levels in LS [14,15]. However, the findings differ from trends in the adult population, showing increasing level of LS across time [1]. Regarding the demographic variables, a significant curvilinear association was found between age and LS, where LS decreased slightly with age between 13 and 18 and increased from the age of 19 to 20 years. Furthermore, boys scored significantly higher on LS than girls when controlled for age, but not when controlled for stress domains and self-rated health. The findings correspond with previous studies showing that socio-demographic factors contribute modestly to adolescents’ reported LS, although variations are normal during adolescence [6,9]. The present study findings of the relatively stable levels of LS are interesting based on previous studies showing an increasing prevalence of self-reported mental health problems during the last decade, especially for girls [10,11], in addition to increasing levels of stress experience [24] and psychosomatic health problems during adolescence [12,36,37].

Of the variables investigated in the present study, all stressor domains were strongly negatively associated with LS; the most strongly related in the multivariate linear regression model were stress due to peer pressure and the home environment and stressors in the school context. Furthermore, significant interaction effects of sex by peer pressure, home life, school attendance, and school performance were found. The strength of the associations was especially strong for girls when controlling for age, time, and self-rated health, indicating that the interpersonal and school-related stressors impact girls’ and boys’ perceptions of LS differently. The present findings of a negative association between stressors and LS are in line with related studies showing that the experience of cumulative and simultaneous stressors, especially those in an interpersonal context, affects adolescents’ mental health and wellbeing [24,38]. The findings are also in line with previous studies showing that stressors in the school context are relevant for adolescents’ LS [14,24].

The present study findings extend prior research by providing insights into the relatively stable level of LS in the adolescent samples investigated across time points. The findings also contribute by showing the significant role of interpersonal and school-related stressors in association with LS when demographic variables and self-rated health are controlled. An implication of the study is to use the results as a knowledge base in strategies to facilitate positive coping in adolescents’ daily life contexts (home/family, community, leisure time, and school). Implementing such strategies relies on cross-sectorial collaboration and integration into different developmental contexts in adolescents’ lives. School is one important context for adolescents’ positive development and wellbeing with the possibility to work on health-promoting approaches. In the school context, it is possible to work on the school climate and the learning environment as well as students’ socio-emotional skills through whole-school approaches, where school professionals and school health services are involved [39].

### Strengths and Limitations

This study was based on three surveys conducted in 2011, 2016, and 2022 in public schools in Mid-Norway using the same previously validated instruments. This allowed us to investigate cross-sectional trends over a 10-year period. The sample size from 2022 was relatively small compared with 2016 and 2011, resulting from COVID-19-related restrictions. Although the same municipalities participated in the three samples, some of the participating schools differed across the sub-samples/study years. Adolescents’ perceptions of LS are likely to be affected by a range of personal and contextual factors; therefore, it is plausible that other variables in addition to those included (e.g., socio-economic status, mental health distress, and personality factors) are equally important in accounting for variance in adolescents’ perceptions of LS. This was a correlational study and causal conclusions cannot be drawn. A longitudinal design would have allowed the investigation of within-person changes in LS over time. Although self-reporting is a well-used method for assessing subjective phenomena in adolescents, it may also present potential challenges with reference to self-report bias (social desirability and over- and under-reporting). However, the large sample size may contribute to protecting from the influences of potential bias related to sample selection and self-report.

## 5. Conclusions

This study supports the relatively high and stable level of LS in adolescents across the time points investigated, although significant differences in LS scores were found between 2011 and 2016. LS decreased slightly between 13 and 18 years old and increased from 19 and 20 years old. Sex differences in LS were found when controlling for age, but not when controlling for time, stress domains, and self-rated health. All stressors were significantly negatively related to LS, with peer pressure and home life showing the strongest association. Significant interaction effects of sex by interpersonal and school-related stressors in association with LS were found, with stronger associations for girls. The results indicate that stressors affect boys’ and girls’ LS differently.

## Figures and Tables

**Table 1:** Demographic characteristics for the adolescent sample aged 13–20 years.

	2011	2016	2022	Total
	n (%)	n (%)	n (%)	N (%)
Sex				
Girls	634 (51.2)	580 (47.0)	167 (53.7)	1381 (49.6)
Boys	603 (48.7)	644 (52.3)	141 (45.3)	1388 (49.9)
Not Specified	2 (0.2)	9 (0.7)	3 (1.0)	14 (0.5)
Age				
13–14	540 (43.6)	147 (11.9)	58 (18.6)	745 (26.8)
15–16	430 (34.7)	371 (30.1)	122 (39.3)	923 (33.2)
17–20	269 (21.7)	715 (58.0)	131 (42.1)	1115 (40.0)
Total	1239 (100)	1233 (100)	311 (100)	2783 (100)

**Table 2:** Mean scores on life satisfaction, stress, and self-rated health across time points.

	2011	2016	2022	Total		
	Mean (SD)				Min/ Max	Cronbach’s α
Life satisfaction (total)	23.11 (6.19)	24.59 (6.20)	22.89 (7.37)	23.77 (6.38)	5–35	0.87
Girls	22.30 (6.06)	23.55 (6.51)	22.60 (7.83)			
Boys	24.04 (6.22)	25.52 (5.76)	23.21 (6.81)			
Age groups						
13–14	23.92 (6.01)	26.62 (5.47)	26.30 (6.31)			
15–16	22.39 (6.54)	24.47 (6.21)	22.66 (7.20)			
17–20	22.84 (6.19)	24.22 (6.27)	21.46 (7.53)			
Stress domains						
Teacher interaction	7.61 (4.12)	6.47 (3.57)	5.36 (2.54)	6.95 (3.85)	4–20	0.86
Peer pressure	11.00 (4.96)	9.57 (4.53)	8.72 (4.02)	10.23 (4.78)	5–25	0.82
Home life	9.97 (4.58)	8.77 (4.06)	7.74 (3.85)	9.29 (4.39	5–25	0.83
Romantic relationships	7.74 (4.73)	6.55 (4.05)	5.57 (3.43)	7.07 (4.43)	4–20	0.85
School attendance	8.45 (3.58)	8.12 (3.41)	7.58 (3.50)	8.24 (3.52)	4–20	0.70
School/leisure conflict	10.39 (4.38)	9.07 (4.06)	8.00 (3.89)	9.64 (4.30)	4–20	0.81
School performance	10.04 (4.05)	9.70 (4.02)	8.65 (3.96)	9.75 (4.05)	4–20	0.83
Self-rated health	3.79 (.97)	3.25 (1.36)	3.80 (1.04)	3.55 (1.20)	1–5	

**Table 3:** Multiple linear regression analysis of associations between sex, age, time, stressor domains, and life satisfaction.

	Life Satisfaction					
	Unadjusted		Adjusted Model ^a^		Adjusted Model ^b^	
	B	β	B	β	B ^b^	β
Sex—girls ref.cat	1.80	0.14 ***	1.83	0.14 ***	0.41	0.03
Age	−0.23	−0.06 ***	−0.25	−0.07 ***	−0.33	−0.09 ***
Age squared	0.08	0.70 *	0.08	0.74 *	0.08	0.04 *
Time dummy 2011 ref.cat						
2016	1.48	0.12 ***	2.07	0.16 ***	0.49	0.04
2022	−0.22	−0.01	0.32	0.02	−0.67	−0.04
Stress domains:						
Teacher interaction (TI)	−0.30	−0.18 ***	−0.30	−0.19 ***	−0.22	−0.14 ***
Peer pressure (PP)	−0.46	−0.34 ***	−0.45	−0.34 ***	−0.36	−0.27 ***
Home life (HL)	0.48	−0.33 ***	−0.47	−0.32 ***	−0.37	−0.26 ***
Romantic relations (RR)	−0.21	−0.14 ***	−0.20	−0.14 ***	−0.16	−0.11 ***
School attendance (SA)	−0.61	−0.34 ***	−0.59	−0.33 ***	−0.42	−0.23 ***
School/leisure conflict (SLC)	−0.32	−0.22 ***	−0.30	−0.20 ***	−0.24	−0.16 ***
School performance (SP)	−0.50	−0.32 ***	−0.48	−0.30 ***	−0.35	−0.23 ***
Sex × TI					0.08	0.05
Sex × PP					0.15	0.13 *
Sex × HL					0.16	0.13 ***
Sex × RR					−0.02	−0.01
Sex × SA					0.17	0.13 *
Sex × SLC					0.11	0.09
Sex × SP					0.13	0.11 ***
Sex × Time					0.01	1.84

Note. Unadjusted analyses present bivariate estimates. ^a^ Model: adjusted for sex/age. ^b^ Model: adjusted for sex, age, time, stress, and self-rated health. Sex: girls—0 and boys—1. Time dummy—2011 is the reference category. Age-squared test curve linearity of age. n = 1943, R^2^ = 0.32; Cohen’s f^2^ = 0.47; * *p* ≤ 0.05; *** *p* ≤ 0.001.

## Data Availability

Not applicable.
